# Evaluating the Effectiveness of Essential Oils and Combination of Copper and Lactic Acid on the Growth of *E. coli* O157:H7 in Laboratory Medium

**DOI:** 10.3390/foods5010014

**Published:** 2016-02-23

**Authors:** Tarik Bor, Rabin Gyawali, Salam A. Ibrahim

**Affiliations:** Food Microbiology and Biotechnology Laboratory, North Carolina Agricultural and Technical State University, Greensboro, NC 27411, USA; tarikbor@gmail.com (T.B.); rgyawali@aggies.ncat.edu (R.G.)

**Keywords:** *E. coli* O157:H7, copper, lactic acid, armoise, clove bud, essential oil

## Abstract

In this study, we compared the effectiveness of armoise and clove bud essential oils (EOs) and the combination of low concentrations of copper (Cu) and lactic acid (LA) against *E. coli* O157:H7 in a laboratory medium. Three strains of *Escherichia coli* O157:H7 (ATCC700599, ATCC51659, and ATCC43895) were used in this study. Antibacterial activity was determined by measuring the turbidity of a broth medium and by determination of bacterial populations. Our results showed that armoise (0.15% *v/v*), clove bud (0.1% *v/v*) EOs, or Cu (50 ppm) in combination with LA (0.2% *v/v*) caused a minimum 5.0 log reduction of *E. coli* O157:H7 in the laboratory medium. Cu in combination with LA may thus be preferable to EOs in food in order to control the growth of foodborne pathogens. In addition, the combination treatment of Cu and LA could provide the food industry with a practical approach to reducing the risk of foodborne pathogens.

## 1. Introduction

Despite advances in food safety, each year roughly 48 million people get sick, 128,000 people are hospitalized and 3000 people die because of foodborne diseases [1]. Due to recent outbreaks of illnesses caused by foodborne pathogens, consumers have been paying more attention to food safety. *E. coli* O157:H7 is one of the major foodborne pathogens that can cause severe illness such as diarrhea and acute kidney failure [1]. Different preservation methods such as pasteurization, irradiation, vacuum packaging and chemical preservatives have been used to control foodborne pathogens [2]. Consumers are not only paying more attention to foodborne pathogens but are also concerned with the use of chemical preservatives to control pathogens in food products [3]. Consumers are demanding minimally processed, safe, shelf-stable foods and prefer natural ingredients [4].

Recently, our laboratory studied the effects of the combination of copper (Cu) and lactic acid (LA) on the growth of *E. coli* O157:H7 and discovered that low concentrations of both Cu and LA could inhibit the growth of foodborne pathogens including *E. coli* O157:H7 and *Salmonella* spp. [5,6]. This approach appears to be very practical for the food industry in order to reduce the risk of foodborne pathogens. Moreover, the combination treatment of Cu and LA is water-soluble and can be a low-cost treatment method compared to treatment with other natural ingredients.

Essential oils (EOs) are common natural preservatives that have been effectively tested against foodborne pathogens. However, the insolubility of EOs in water and their strong aroma could negatively impact the sensory perception of consumers and thus limit the use of EOs in food products [7]. Previous studies in our laboratory showed that low concentrations of Cu and LA could achieve a 5 log reduction of *E. coli* O157:H7 [5,6]. However, there is a need to conduct a study to validate the effectiveness of low concentrations of copper and lactic acid as antimicrobials with other known natural ingredients such as EOs. Therefore, the objective of this study was to evaluate the effectiveness of EOs (armoise and clove oils) and the combination of Cu and LA to control the growth of *E. coli* O157:H7 in a laboratory medium.

## 2. Experimental Section

### 2.1. Bacterial Strains and Inoculm Prpearation

Three strains of *E. coli* O157:H7, ATCC 700599 (salami isolate), ATCC 51659 (clinical isolate), and ATCC 43895 (raw hamburger meat isolate), were individually grown in BHI (Difco, Becton Dickinson, Sparks, MD, USA) at 37 °C for 24 h. Overnight cultures of the strains were centrifuged (8000 *g*, 15 min at 4 °C), supernatant of each strain was decanted and the cell pellet was re-suspended (approximately 8.0 log CFU/mL) in 0.1% (*v/v*) 10 ml peptone water. Individual strains were prepared by mixing 1 mL of each overnight strain in 9.0 mL of peptone water. Several decimal dilutions were then made to achieve an initial inoculum level of 2–3 log CFU/mL.

### 2.2. Experimental Design

Armoise (*Artemisia alba*, armoise EO, Batch No. 1444, Origin: Morocco, Aromatics Inc., Ontario, Canada) and clove bud (*Syzygium aromaticum*, Organic clove bud oil, Origin: Madagascar, Simplers Botanicals) EOs were purchased from local stores in Greensboro, NC, and kept at 4 °C for 24 h. EOs were mixed with sterilized Tween 80 (1:1) (Fisher Scientific, Pittsburgh, PA, USA) and added into sterilized BHI broth to obtain 0.006%, 0.0125%, 0.025%, 0.05%, 0.1%, 0.15%, 0.2%, and 0.25% (*v/v*) concentrations. Seven mL batches of samples from each portion were dispensed into sterile test tubes. Seven mL of BHI broth without EOs were used as control samples. Along with the treatment samples, a blank sample containing the same treatment concentrations were used to correct the sample readings. Each set of experimental tests was conducted in triplicates.

The antimicrobial activity of low-concentration Cu and LA was tested according to the method described by Gyawali *et al.* [5]. In this procedure, 7.0 mL batches of fresh BHI broth containing copper sulfate pentahydrate (CuSO_4_.5H_2_O, Thermo Fisher Scientific, Fair Lawn, NJ, USA) at concentrations of 25 and 50 ppm (*w/v*), LA at 0.1%, 0.15% and 0.2% (*v/v*) (85%, Thermo Fisher Scientific, Fair Lawn, NJ, USA) and their combinations were prepared. An additional 7.0 mL of BHI broth that was not supplemented with any chemical was used as a control. Samples containing the control and LA were autoclaved at 121 °C for 15 min. The Cu solution was filter sterilized through a 0.2 µm Nalgene filtration product (Nalge Nunc International, Rochester, NY, USA) and added to the BHI broth at varying concentrations.

### 2.3. Measuring Bacterial Growth

Bacterial growth was monitored by measuring turbidity at two-hour time intervals during incubation at 37 °C over 8 h using a GENESYS™ 10S UV-Vis spectrophotometer (Thermo Electron Scientific Co., Madison, WI, USA) at a 610 nm wavelength.

### 2.4. Bacterial Enumeration

Bacterial populations were determined by plating onto BHI agar. In this procedure, samples (1 mL) were withdrawn from inoculated samples at the beginning (0 h) and end (8 h) of the incubation period and serially diluted in 0.1% peptone water. Then, appropriate dilutions were surface-plated (100 µL) onto duplicate BHI agar and colonies were counted after plates were incubated at 37 °C for 24 h.

### 2.5. Statistical Analysis

Different treatments were statistically analyzed for their antimicrobial effects on *E. coli* O157:H7 by factorial analysis of variance of duplicate samples. Each experiment was conducted in replicates to determine the effects of Cu and LA combination treatment, armoise EO and clove bud EO on the survival and growth of the bacterial strains. Data from the three experiments were pooled to calculate the statistical mean and standard error of the mean for each treatment. Data were verified for normality, followed by an analysis of variance (*p* < 0.05) using SAS version 9.2 (SAS Institute, Cary, NC, USA).

## 3. Results and Discussion

### 3.1. Antibacterial Activity of Armoise and Clove Bud EOs against *E. coli* O157:H7 in BHI Medium

**Figure 1:**
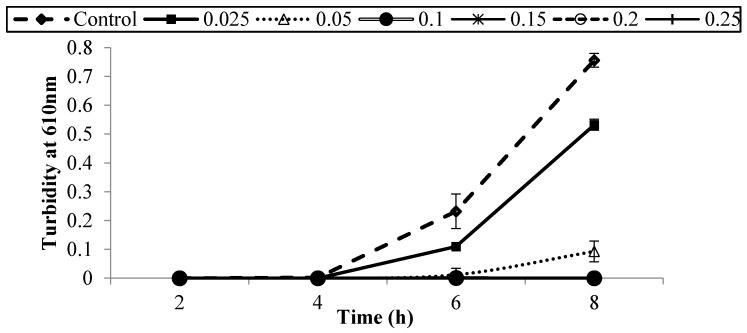
Survival and growth of *E. coli* O157:H7 (ATCC 700599) in BHI medium with different concentrations of armoise EO (%, *v/v*) after 8 h incubation at 37 °C. Symbols represent means of triplicate replications (*n* = 3); error bars depict standard deviation from the sample mean.

**Figure 2:**
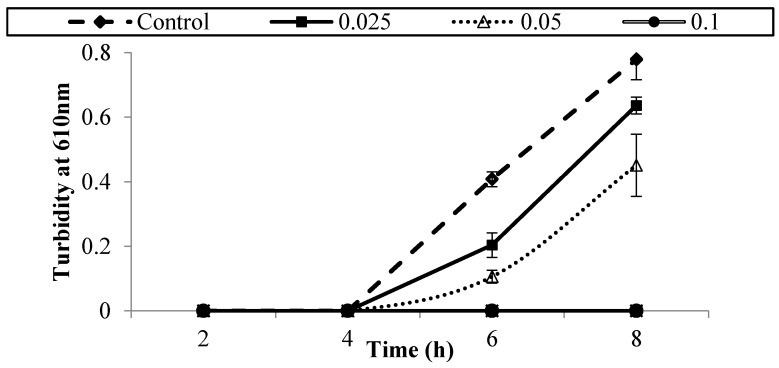
Survival and growth of *E. coli* O157:H7 (ATCC 51659) in BHI medium with different concentrations of armoise EO (%, *v/v*) after 8 h incubation at 37 °C. Symbols represent means of triplicate replications (*n* = 3); error bars depict standard deviation from the sample mean.

**Figure 3:**
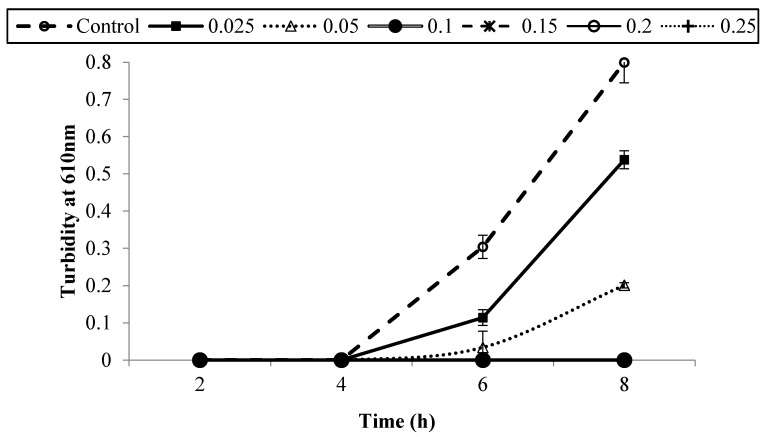
Survival and growth of *E. coli* O157:H7 (ATCC 43895) in BHI medium with different concentrations of armoise EO (%, *v/v*) after 8 h incubation at 37 °C. Symbols represent means of triplicate replications (*n* = 3); error bars depict standard deviation from the sample mean.

**Figure 4:**
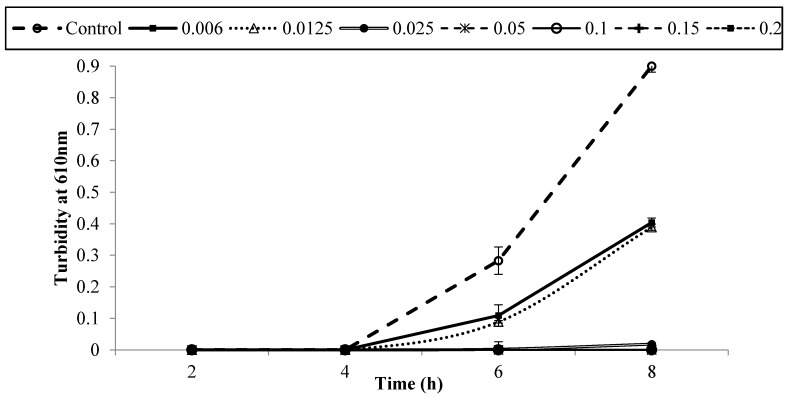
Survival and growth of *E. coli* O157:H7 (ATCC 700599) in BHI medium with different concentrations of clove bud EO (%, *v/v*) during incubation at 37 °C for 8 h. Symbols represent means of triplicate replications (*n* = 3); error bars depict standard deviation from the sample mean.

**Figure 5:**
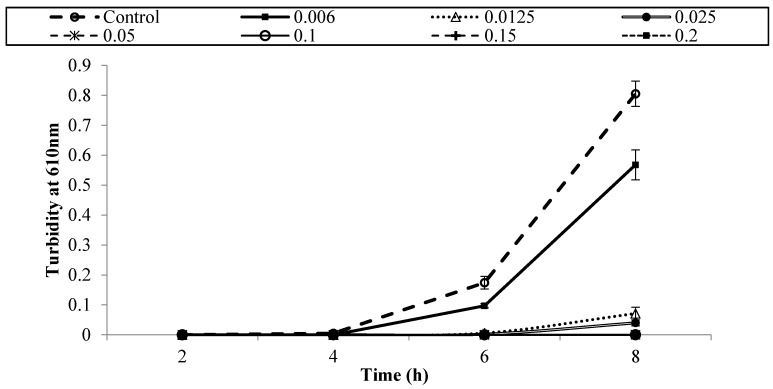
Survival and growth of *E. coli* O157:H7 (ATCC 51659) in BHI medium with different concentrations of clove bud EO (%, *v/v*) during incubation at 37 °C for 8 h. Symbols represent means of triplicate replications (*n* = 3); error bars depict standard deviation from the sample mean.

**Figure 6:**
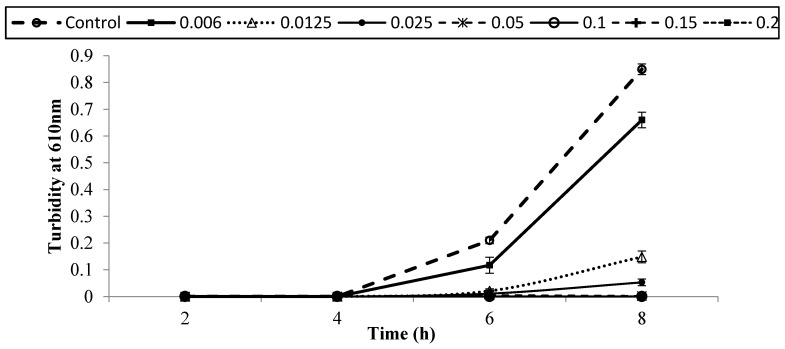
Survival and growth of *E. coli* O157:H7 (ATCC 43895) in BHI medium with different concentrations of clove bud EO (%, *v/v*) during incubation at 37 °C for 8 h. Symbols represent means of triplicate replications (*n* = 3); error bars depict standard deviation from the sample mean.

Figure 1, Figure 2, Figure 3, Figure 4, Figure 5 and Figure 6 show survival and growth of three strains of *E. coli* O157:H7 (ATCC700599, ATCC51659, and ATCC43895) in BHI medium with different concentrations of armoise and clove bud EOs during incubation at 37 °C for 8 h. In the control samples, the initial growth as determined by turbidity at 610 nm was ~0.0. The bacterial strains continued to grow during the incubation period and reached the stationary phase within 6–8 h. Turbidity readings reached ~0.80 after 8 h. With the addition of armoise EO at low concentrations (0.025% and 0.05%), turbidity readings were similar to the control samples, while high concentrations of armoise EO (≥0.1%) inhibited the growth of all tested strains as evidenced by low turbidity readings. Turbidity readings were ~0.50 with the addition of clove bud EO at low concentrations (0.006%), and ~0.0 at high concentrations (≥0.0125).

At the end of the incubation period, the population of all tested strains was determined (Table 1 and Table 2). In control samples, the population of *E. coli* O157:H7 increased from ~2–3 log CFU/mL of initial population to ~8.8 log CFU/mL after 8 h. With the addition of armoise and clove bud EOs at low concentrations (≤0.0125%), growth was similar to the control samples; however, at high (≥0.15%) concentrations the population of all tested strains was <1 log CFU/mL. Based on turbidity readings and populations of tested strains, our results indicated that the armoise and clove bud EOs at 0.1% (*v/v*) inhibited the growth of all tested strains in BHI broth medium. These results are supported by Imelouane *et al.* [8], who showed that *Artemisia herba-*Alba Asso. EO at a 0.66 mg/mL concentration inhibited the growth of *E. coli* O157:H7 in TSB. Similarly, Friedman *et al.* [9] found that clove bud EO exerted bactericidal activity against *E. coli* O157:H7 when tested in an aqueous phosphate-saline buffer (pH 7.0).

### 3.2. Antibacterial Activity of Copper (Cu) and Lactic Acid (LA) against E. coli O157:H7 in BHI Medium

**Figure 7:**
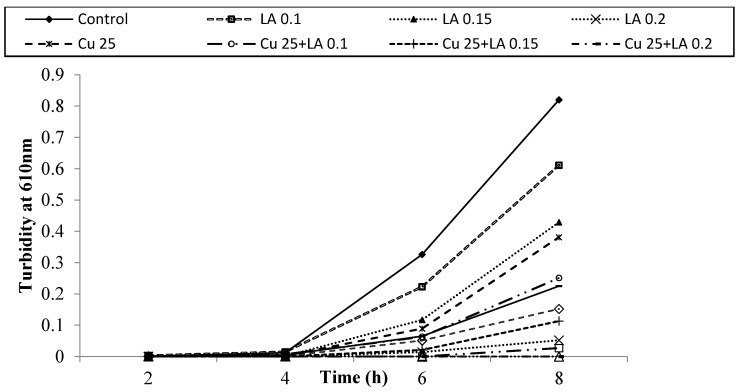
Survival and growth of *E. coli* ATCC 700599 in BHI broth with copper (ppm, *w/v*) and lactic acid (% *v/v*) at different concentrations during incubation at 37 °C for 8 h.

**Figure 8:**
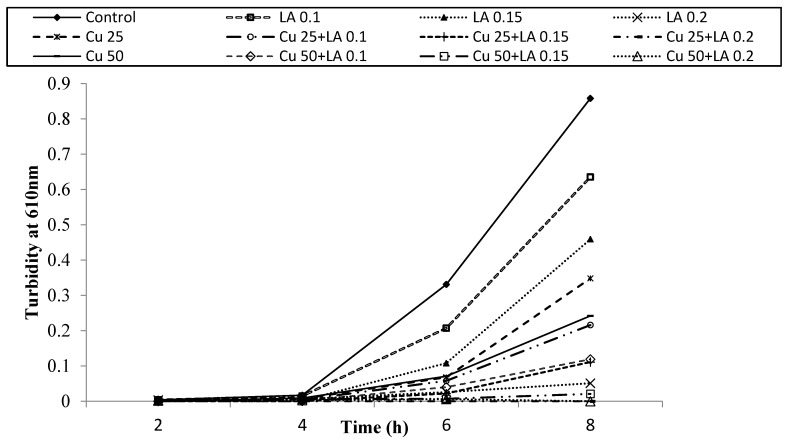
Survival and growth of *E. coli* ATCC 51659 in BHI broth with copper (ppm, *w/v*) and lactic acid(% *v/v*) at different concentrations during incubation at 37 °C for 8 h.

**Figure 9:**
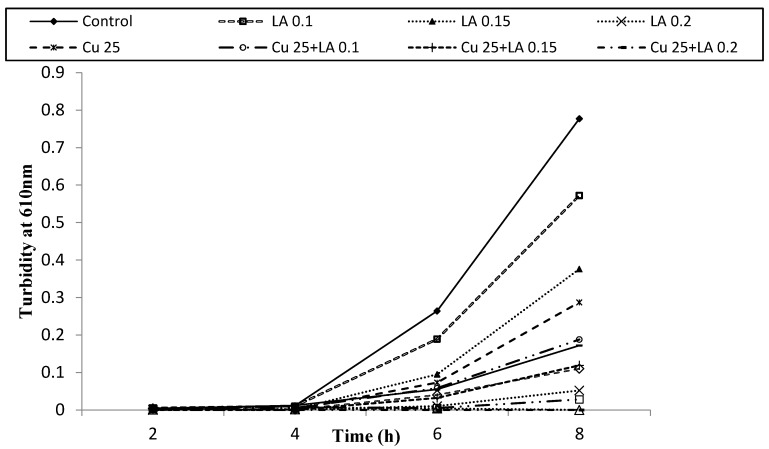
Survival and growth of *E. coli* ATCC 43895 in BHI broth with copper (ppm, *w/v*) and lactic acid (% *v/v*) at different concentrations during incubation at 37 °C for 8 h.

Figure 7, Figure 8 and Figure 9 show survival and growth of *E. coli* O157:H7 strains (ATCC 700599, ATCC 51659 and ATCC 43895) in the presence of Cu and LA at different concentrations during incubation at 37 °C for 8 h. The bacterial strains continued to grow during the incubation period and reached the stationary phase within 6–8 h. The turbidity readings reached an absorbance of ~0.82. When Cu was added to BHI broth at a concentration of 25 ppm, absorbance was ~0.34, and at a concentration of 50 ppm Cu, absorbance was ~0.21. The growth of bacterial strains was similar to the control with the addition of LA at concentrations of 0.1% and 0.15%. However, when a combination of Cu (25 and 50 ppm) and LA (0.1% and 0.15%) was used, the turbidity reading was 0.0.

Table 3 shows the population of *E. coli* O157:H7 in the presence of different concentrations of Cu and LA after incubation at 37 °C for 8 h. In the control samples, the population of *E. coli* O157:H7 increased from ~2.0 to 3.0 log CFU/mL to ~9.0 log CFU/mL after 8 h. Cu or LA alone slightly inhibited the growth of tested strains (~2.0 log reduction). The combination of Cu (25 and 50 ppm) with LA (0.1% and 0.15%) caused a ~4.0 log reduction, while Cu 50 ppm and LA 0.2% caused a ~7.0 log reduction on the growth of *E. coli* O157:H7. Our results indicated that Cu and LA alone had a slight effect on the growth of *E. coli* O157:H7 in the laboratory medium. The combination of Cu and LA showed significant growth inhibition for all tested strains (*p* < 0.05). Al-Holy *et al.* [10] investigated the effect of LA, Cu (II), and monolaurin as natural antimicrobials against *Cronobacter* in infant formula. These authors showed that a complete elimination of *Cronobacter* was obtained when a combination of sub-lethal concentrations of LA (0.2%) and Cu (II) (50 ppm) was used. The results indicate that Cu and LA could be used to control the growth of a wide range of pathogens.

The Food and Drug Administration (FDA) requires food processors to achieve at least a 5.0 log reduction of pathogens in their finished products [11]. This ruling has prompted the search for other novel non-thermal preservation methods that can ensure product safety. In our study, we have demonstrated the combination treatment of Cu and LA as a potentially promising non-thermal food preservation method which meets the US FDA’s requirement of a mandatory 5.0 log reduction. The synergistic effect of Cu and LA in carrot juice samples was shown in our previous studies [6]. In our current study, we demonstrated that armoise and clove bud oils at certain concentrations reduced the number of tested strains by at least 5 log in laboratory medium. We also showed that Cu and LA at low concentrations caused at least a 5 log reduction of tested strains. In recent years, there has been an increased interest in the use of natural antimicrobial agents, especially Eos, to control foodborne bacteria and other pathogenic microorganisms. EOs have antibacterial and antioxidant properties and can be used in different applications to ensure food safety [12]. However, EOs are expensive and are not water-soluble. To achieve a 5.0 log reduction, EOs need to be added above the solubility limit, and that could have an impact on the flavor and appearance of the food and affect overall sensory properties. Cu at a low concentration is relatively safe and non-toxic for humans and is considered an essential micronutrient and cofactor for certain enzymes [6].

Due to its strong antimicrobial effect at low concentrations, low cost of chemicals, lack of negative impact on sensory properties, lack of binding with lipids and protein components, and due to its water solubility properties, Cu in combination with LA would be superior to EOs at inhibiting the growth of microorganisms to ensure the safety of food products.

## 4. Conclusions

Our study revealed that armoise (0.15% *v/v*) and clove bud (0.1% *v/v*) EOs caused a 5.0 log reduction in the growth of *E. coli* O157:H7 in BHI medium. When both Cu and LA were added at low concentrations into BHI broth, the growth of all tested strains of *E. coli* O157:H7 was significantly inhibited (*p* < 0.05). These results clearly indicate that both EOs and a combination of Cu and LA can be used to achieve a 5.0 log reduction of pertinent pathogens. However, Cu and LA at low concentrations could be a practical alternative to EOs in order to achieve 5.0 log reductions in food products for industrial applications. Further studies are needed to better understand the effectiveness of Cu and LA on the growth of *E. coli* O157:H7 in different food models.

## Figures and Tables

**Table 1:** Population of *E. coli* O157:H7 in Brain Heart Infusion (BHI) broth medium in the presence of armoise essential oil (EO) at different concentrations after incubation at 37 °C for 8 h.

Treatment (% *v/v*)	Population of *E. coli* O157:H7 Strains (log CFU/mL)		
	ATCC 700599	ATCC 51659	ATCC 43895
Control	9.01 ± 0.11 ^a^	8.82 ± 0.11 ^a^	8.41 ± 0.53 ^a^
EO 0.025	8.26 ± 0 ^b^	8.68 ± 0.26 ^a^	8.22 ± 0.29 ^b^
EO 0.05	8.1 ± .005 ^b^	7.96 ± 0.18 ^b^	6.93 ± 0.28 ^c^
EO 0.1	<1 ^c^	2.34 ± 0.19 ^c^	<1 ^d^
EO 0.15	<1 ^c^	<1 ^d^	<1 ^d^
EO 0.2	<1 ^c^	<1 ^d^	<1 ^d^
EO 0.25	<1 ^c^	<1 ^d^	<1 ^d^

Initial inoculum level was 2–3 log CFU/mL. Statistically different treatments (*p* < 0.05) within same column are shown with different letters. Values (log CFU/mL) are mean ± standard deviation (*n* = 3).

**Table 2:** Population of *E. coli* O157:H7 with the presence of clove bud essential oil (EO) at different concentrations (% *v/v*) in Brain Heart Infusion (BHI) broth medium after incubation at 37 °C for 8 h.

Treatment (% *v/v*)	Population of *E. coli* O157:H7 Strains (log CFU/mL)		
	ATCC 700599	ATCC 51659	ATCC 43895
Control	8.48 ± 0.05 ^a^	8.52 ± 0.47 ^a^	7.23 ± 0.26 ^a^
EO 0.006	7.71 ± 0.04 ^b^	7.22 ± 0.35 ^b^	6.74 ± 0.13 ^a^
EO 0.0125	7.06 ± 0.02 ^c^	5.2 ± 0.14 ^c^	4.66 ± 0.19 ^b^
EO 0.025	3.06 ± 0.41 ^d^	4.6 ± 0.11 ^d^	4.23 ± 0.59 ^b^
EO 0.05	2.27 ± 0.04 ^e^	1.94 ± 0.02 ^e^	1.89 ± 0.01 ^c^
EO 0.1	<1 ^f^	<1 ^f^	<1 ^d^
EO 0.15	<1 ^f^	<1 ^f^	<1 ^d^
EO 0.2	<1 ^f^	<1 ^f^	<1 ^d^

Initial inoculum level was 2–3 log CFU/mL. Statistically different treatments (*p* < 0.05) within same column are shown with different letters. Values (log CFU/mL) are mean ± standard deviation (*n* = 3).

**Table 3:** Population of *E. coli* O157:H7 in BHI medium in the presence of lactic acid (LA) at 0.1%, 0.15%, and 0.2% *v/v* and copper (Cu) at 25 and 50 ppm concentrations after incubation at 37 °C for 8 h.

Treatment	Population of *E. coli* O157:H7 Strains (log CFU/mL)		
	ATCC 700599	ATCC 51659	ATCC 43895
CONTROL	8.79 ± 0.21 ^a^	8.70 ± 0.25 ^a^	8.74 ± 0.23 ^a^
LA 0.1	8.55 ± 0.23 ^a^	8.18 ± 0.56 ^ab^	8.35 ± 0.19 ^ab^
LA 0.15	8.1 ± 0.30 ^ab^	7.46 ± 0.27 ^bc^	6.55 ± 0.16 ^d^
LA 0.2	5.69 ± 0.08 ^e^	5.49 ± 0.38 ^ef^	6.10 ± 0.41 ^de^
Cu 25 ppm	7.62 ± 0.14 ^bc^	6.98 ± 0.63 ^cd^	7.77 ± 0.32 ^bc^
Cu 25 + LA 0.1	6.98 ± 0.18 ^c^	6.40 ± 0.20 ^cde^	7.28 ± 0.41 ^c^
Cu 25 + LA 0.15	6.01 ± 0.48 ^de^	6.18 ± 0.27 ^de^	5.46 ± 0.23 ^ef^
Cu 25 + LA 0.2	4.58 ± 0.26 ^f^	4.92 ± 0.21 ^f^	4.58 ± 0.26 ^g^
Cu 50	7.16 ± 0.61 ^c^	6.98 ± 0.81 ^bd^	6.47 ± 0.15 ^d^
Cu 50 + LA 0.1	6.79 ± 0.40 ^cd^	5.31 ± 1.19 ^ef^	5.01 ± 0.47 ^fg^
Cu 50 + LA 0.15	5.28 ± 0.49 ^ef^	4.80 ± 0.10 ^f^	3.24 ± 0.15 ^h^
Cu 50 + LA 0.2	1.61 ± 0.67 ^g^	1.50 ± 0.92 ^g^	0.84 ± 0.20 ^i^

Initial inoculum level was 2–3 log CFU/mL. Statistically different treatments (*p* < 0.05) within same column are shown with different letters. Values (log CFU/mL) are mean ± standard deviation (*n* = 3).
